# Outcomes of mid-term and long-term degradable biosynthetic meshes in single-stage open complex abdominal wall reconstruction

**DOI:** 10.1007/s10029-021-02415-7

**Published:** 2021-06-07

**Authors:** J. J. M. Claessen, A. S. Timmer, J. J. Atema, M. A. Boermeester

**Affiliations:** Department of Surgery, Amsterdam Gastroenterology and Metabolism, Infection and Immunity, Amsterdam UMC, Location AMC, University of Amsterdam, Amsterdam, The Netherlands

**Keywords:** Biosynthetic mesh, CAWR, mid-term degradable, long-term degradable, Bio-A, Phasix

## Abstract

**Objective:**

To assess clinical outcomes in patients that underwent open single-stage complex abdominal wall reconstruction (CAWR) with biosynthetic mesh.

**Methods:**

Retrospective observational study of two prospectively registered series of consecutive patients undergoing CAWR with either long-term degradable (LTD) Phasix^™^ or mid-term degradable (MTD) BIO-A^®^ biosynthetic mesh in a single institution between June 2016 and December 2019.

**Results:**

From 169 patients with CAWR, 70 consecutive patients were identified who underwent CAWR with either LTD or MTD biosynthetic mesh. More than 85% of patients had an incisional hernia that could be classified as moderately complex to major complex due to a previous wound infection (67%), one or more complicating comorbidities (87.1%), one or more complicating hernia characteristics (75.7%) or contaminated or dirty defects (37.1%). Concomitant component separation was performed in 43 of 70 patients (61.4%). Overall surgical site infection (SSI) rate in these CAWR patients was 45.7%. Seventeen of 70 patients (24.3%) had computed tomography (CT) - and culture-confirmed SSI in direct contact of mesh, suspicious of mesh infection. Mesh removal for persistent local infection occurred in 10% (7 of 70) after a median of 229 days since surgery. Salvage rate of mesh after direct contact with infection was 58.8%. All removed meshes were in the LTD group. Seven patients (10%) had a recurrence; four patients in the LTD group (10%) had a recurrence at a median follow-up of 35 months and three patients in the MTD group (10%) at a median follow-up of 11 months. Three of the seven recurrences occurred in patients with SSI in persistent and direct contact with mesh.

**Conclusions:**

Comorbid patients undergoing open complex abdominal wall reconstruction are at high risk of postoperative wound complications regardless of which type of biosynthetic mesh is used. When in persistent and direct contact with infection, long-term biodegradable biosynthetic meshes may need to be removed, whereas mid-term biodegradable biosynthetic meshes can be salvaged.

**Supplementary Information:**

The online version contains supplementary material available at 10.1007/s10029-021-02415-7.

## Introduction

Ventral hernia development is one of the most common complications following laparotomy [1]. Where suture repair is associated with unacceptably high recurrence rates, the use of synthetic mesh has acceptable results following abdominal wall reconstruction (AWR) [2]. Patient comorbidities and reconstructions in contaminated hernia sites are associated with postoperative wound complications [3]. The risk of surgical site infection (SSI) and (chronic) mesh infection with the possible need for mesh removal has resulted in surgeons’ reluctance to use permanent synthetic mesh in high-risk settings.

Biologic mesh serves as a temporary scaffold, facilitating revascularization and remodeling of the native abdominal wall [4]. It is proposed that these meshes can withstand or reduce bacterial contamination. Multiple studies investigating the use of biologic mesh in high-risk patients and/or contaminated hernia repair found that most postoperative wound complications can indeed be managed conservatively, and the number of patients that develop mesh infection requiring removal is as low as 0% in non-crosslinked biologic mesh [3, 5–7]. However, reported hernia recurrence rates could be as high as 30% after 3 years [7, 8]. These results combined with the high purchase costs of biologic meshes have led to a search in alternative meshed to be used in complex AWR (CAWR).

Biosynthetic mesh, also known as biodegradable or bioabsorbable mesh, is composed of absorbable synthetic material and aims to provide the same advantages as biologic mesh, providing a more durable repair but at lower cost. Currently, three biosynthetic meshes are available on the market: BIO-A^®^ (Gore, Flagstaff, AZ, USA), Phasix^™^ mesh (CR Bard Inc., Murray Hill, NJ, USA), and TIGR Matrix^™^ (Novus Scientific, Uppsala, Sweden). All three are fully resorbable through hydrolyzation in 7, 18 and 36 months, respectively. Published data on biosynthetic meshes used in CAWR is limited [9–14], and it remains uncertain how these meshes perform when used in high-risk patients and contaminated fields or when in contact with a postoperative SSI. Whether native tissue ingrowth into the temporary scaffold provided by a biosynthetic mesh results in high-quality support to the reconstruction is also in need of more clinical data.

The aim of this study is to assess clinical outcomes in high-risk patients that underwent open single-stage CAWR with one of two types of biosynthetic mesh: a long-term degradable and a mid-term degradable biosynthetic mesh.

## Methods

### Study design

In this observational study, we retrospectively analyzed two prospectively registered series of consecutive patients in which a biosynthetic mesh, either a long-term degradable (LTD) (Phasix^™^, CR Bard Inc.) or mid-term degradable (MTD) (BIo-A^®^, Gore) was used for single-stage open CAWR in the Amsterdam UMC, location AMC, The Netherlands, between June 2016 and December 2019. The study was approved by the Institutional Review Board, and is reported following the Strengthening the Reporting of Observational Studies in Epidemiology (STROBE) statement [15].

### Inclusion criteria

Patients were eligible for inclusion if they underwent an open single-stage CAWR using biosynthetic mesh and consented to the use of their data.

### Data items

Baseline demographic data included age, gender, body mass index (BMI), smoking status, diabetes mellitus (DM), cardiac disease, chronic obstructive pulmonary disease (COPD), use of medication, number of previous abdominal surgeries and abdominal wall reconstructions, presence of stomata, intestinal fistulas and infected mesh, wound classification status according to the Centers for Disease and Control and Prevention [16], preoperative botulinum toxin A (BTA) injections, hernia width, and loss of domain measured as described by Sabbagh et al. [17]. The risk of postoperative complications and hernia recurrence were classified according to the modified Ventral Hernia Working Group grading system (mVHWG) and the hernia, patient, wound (HPW) classification [18, 19]. The mVHWG classification system does not incorporate important risk factors as hernia width and loss of domain. The HPW classification includes hernia width but not loss of domain. To better describe the complexity of patients and their abdominal wall defects, we, therefore, assigned them to one of three severity classes (minor, moderate and major complex) as described by an expert consensus group in 2014 [20].

Operative data included concomitant bowel surgery, use of a component separation technique (CST; anterior CST or transverse abdominis release (TAR)), specific mesh type, mesh location, full-thickness skin reconstruction, incisional negative pressure wound therapy (iNPWT), and posterior and anterior fascial closure. Anterior component separation (ACS) was defined as an open or endoscopic release of the aponeurosis of the external oblique muscle, 1.5 cm lateral to the linea semilunaris. TAR was defined as separation of the transversus abdominis muscle medial of the neurovascular bundles and the linea semilunaris (following a Rives–Stoppa approach) and further dissection over the transverse fascia. Preparation over the posterior rectus sheath with mesh placement in Rives–Stoppa was not scored as a CST.

### Outcome variables

Clinical outcomes analyzed were incidence of (suspected) mesh infection and subsequent need for mesh removal for persistent infection during follow-up, salvage rate of mesh, surgical site occurrence (SSO), SSI, length of hospital stay, reoperations and hernia recurrence. Suspected mesh infection was defined as computed tomography (CT)- and culture- confirmed direct contact of mesh with SSI. Surgical site infection was divided into superficial, deep and organ space according to CDC criteria [16]. Recurrence was assessed during visits to the outpatient clinic and subsequently confirmed with CT. Furthermore, all patients were contacted by telephone and asked if they had visited any other hospital for diagnostics or treatment of their abdominal wall. Patients who did not perceive a recurrence, and declared absence of bulging and pain at the site of the repair, were defined as recurrence free, as a negative reply to these questions has a negative predictive value of 94% for hernia recurrence [21].

Due to the COVID-19 pandemic, we did not invite patients who reported symptoms in line with a possible recurrence for clinical assessment. For patients who deceased during the study period, follow-up extended to the last outpatient visit.

### Analysis

All data items are summarized for the entire group, and in subgroups according to the specific type of biosynthetic mesh used. Numerical data are expressed as mean and standard deviation (SD) or median and range. Categorical data are summarized as count and percentage. Preoperative and operative data were compared using the unpaired *t* test and Chi square or Fisher exact test for numerical and categorical data respectively, with a significant level *p* < 0.05.

**Fig. 1:**
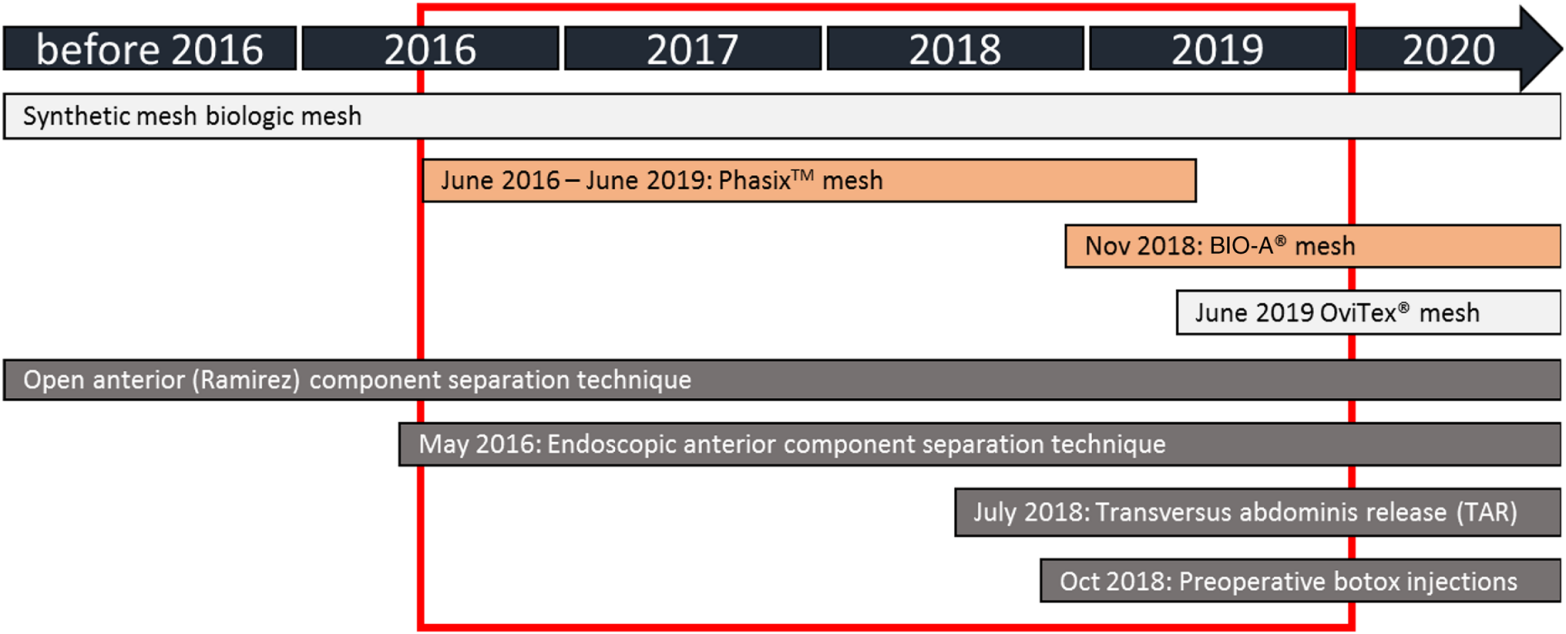
Developments in CAWR in the Amsterdam UMC, location AMC

Due to differences in mesh choice based on patient and hernia characteristics over time (Fig. 1), the LTD and MTD mesh groups are two independent series of consecutive patients. In 2019, in more contaminated or major complex cases, we started to use Ovitex^®^ reinforced tissue matrix, a hybrid biological mesh (TELA Bio, Malvern, PA, USA), and we used the MTD biosynthetic mesh BIO-A^®^ for mild or moderately contaminated hernia repair and without risk of needing a bridging repair. Only further forward in time did we use MTD biosynthetic mesh for more complex cases. Therefore, the LTD and MTD biosynthetic mesh groups are a priori not comparable on hernia and surgery characteristics, and as such postoperative outcomes were not statistically compared between the two mesh groups. However, we did assess the cumulative recurrence rate during follow-up using Kaplan–Meier analysis (log rank). The incidence between both groups is compared at the time point at which one-third of patients from one cohort was still at risk of recurrence (censored analysis).

### Treatment strategy

The tailored treatment differed between patients, but in general treatment strategy was as follows. All patients were preoperatively assessed and optimized by a multidisciplinary team. In short; patients with intestinal failure were optimized according our in-house protocol supervised by our intestinal failure team [22, 23]. Patients with a complex abdominal wall defect and identified risk factors (e.g., smoking, obesity, diabetes, malnutrition, COPD) were optimized during a prehabilitation period before surgery. As of October 2018, patients with defect widths over 10 cm and/or ≥ 20% loss of domain were treated with preoperative BTA injections bilateral in the lateral abdominal wall musculature.

Mesh choice depended on the complexity of the patients and the reconstruction, as well as the type of mesh available at that time period. A timeline of the developments in CAWR approach in our hospital is presented in Fig. 1. Two types of biosynthetic mesh were introduced in our center for more complex patients; since June 2016 a LTD biosynthetic mesh (Phasix^™^) and since November 2018 a MTD biosynthetic mesh (BIO-A^®^).

In patients with clean defects, biosynthetic mesh was used as single mesh repair. In large and contaminated/dirty defects, a dual layer technique was used when the anterior fascia could not be closed despite a CST. This technique comprised an intra-abdominal (underlay) biologic mesh as a leverage with parachuting transfascial sutures to pull close the abdominal cavity and thereby protecting the intra-abdominal viscera; a biosynthetic mesh positioned as retro-rectus (sublay) reinforcement ventral to the biologic mesh creating a dual layer repair.

Superficial wound infections were treated conservatively. Deep and organ space SSIs were treated by radiological percutaneous drainage with or without antibiotics. Percutaneous drainage was also performed when a SSI was in direct contact with mesh, CT and culture confirmed, to salvage the suspected infected mesh. If the mesh location remained persistently infectious, operative placement of a negative pressure wound therapy with instillation (NPWT-I) (V.A.C. VERAFLO^™^, KCI, San Antonio, TX, USA)) was performed. When, despite this treatment, the mesh could not be salvaged during the course of several months of follow-up it was surgically removed.

### Surgical technique

All operative procedures were tailored to the patient; however, all operations were performed in consistence with international guidelines and consensus [24]. The abdomen was encountered through the previous laparotomy incision, transecting and dissecting but not removing the hernia sac, followed by extensive adhesiolysis. Bioburden was reduced by resection of previously placed mesh and non-viable tissue. Enterocutaneous or enteroatmospheric fistulas were resected by segmental bowel resection, and a hand-sewn anastomosis was constructed. Up-stream diverting stomas were used infrequently when necessary. Tension-free midline closure of both fascial layers was attempted with mesh reinforcement, preventing a bridged repair whenever possible. When indicated and feasible, component separation techniques were used. If the posterior fascia could not be brought together in the midline, one side of the hernia sac was used to close the abdominal cavity. If the anterior fascia could not be closed without midline tension, or when both fascial layers could not be closed at all, a double layer mesh technique was used (see “Treatment strategy”). Biosynthetic mesh was preferably placed in the retro-rectus (sublay) plane. Subcutaneous drains were placed to the surgeon’s discretion (predominantly on the mesh and when a CST was used). Prophylactic closed incision negative pressure wound therapy (NPWT; Prevena^™^, KCI, San Antonio, TX, USA)] for 5–7 days at − 125 mmHg has been standard care in our practice since 2014. In the presence of significant loss of skin quality, large full-thickness skin defects and significant loss of domain, reconstruction was performed in collaboration with plastic and reconstructive surgeons.

Postoperatively all patients were instructed to wear an abdominal binder 24/7 during the first 2 weeks and subsequent for 3 months when mobilizing.

## Results

### Participant selection and preoperative data

**Fig. 2:**
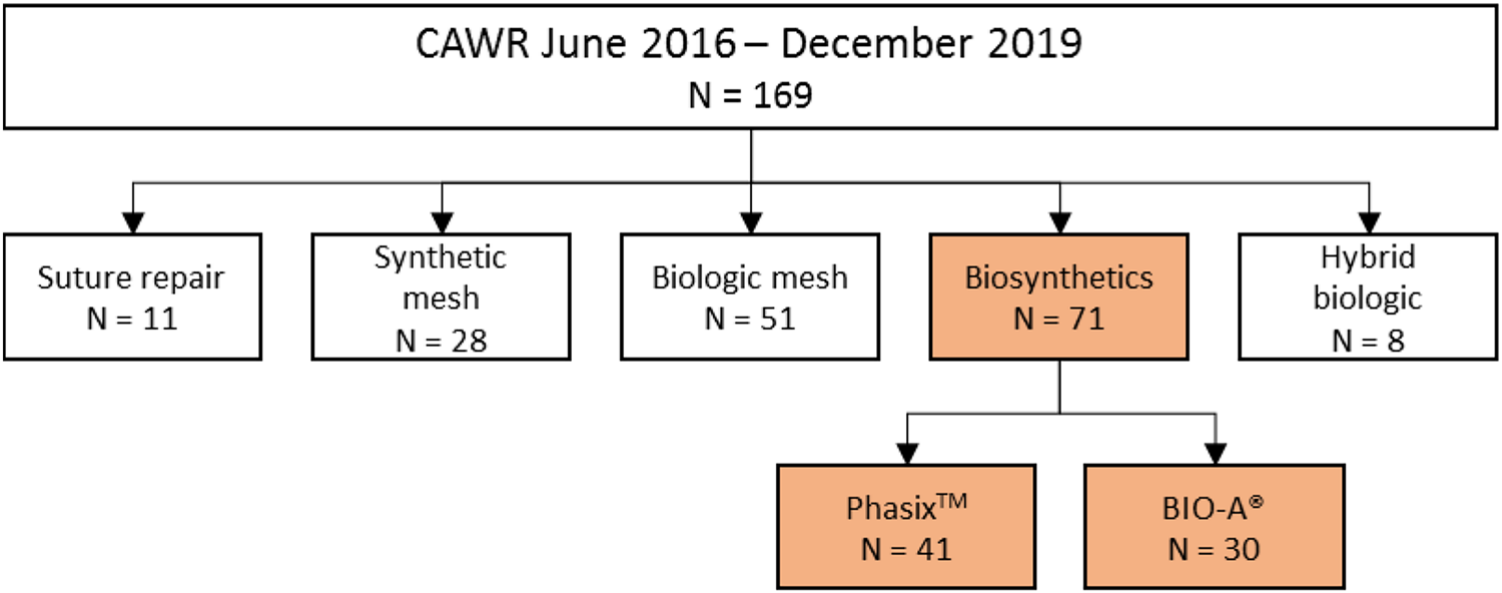
Meshes used of all CAWR between June 2016 and Dec 2019

A total of 169 CAWRs were performed during the study period (June 2016 and December 2019); Fig. 2 depicts the subgroups of different meshes used for CAWR. For types and combinations used during the study period (see Online Supplementary material). In 71 of the 169 reconstructions, biosynthetic mesh was used. One patient did not give informed consent. Therefore, a total of 70 patients were included; 40 patients with LTD biosynthetic mesh, and 30 with MTD biosynthetic mesh.

Baseline patient and hernia characteristics of all CAWRs performed with biosynthetic mesh in the study period are presented in Table 1. More than 85% of patients had an incisional hernia that could be classified as moderate to major complex due to a previous wound infection (67%), one or more complicating comorbidities (87.1%), one or more complicating hernia characteristics (75.7%) or contaminated or dirty defects (37.1%). Sixty-seven percent of patients had experienced a previous SSI prior to their indexed CAWR at our institute. Only three patients were had a mVHWG grade I hernias.

**Table 1:** Preoperative data

	Total group (*n* = 70)	LTD (*n* = 40)	MTD (*n* = 30)	*p *value
	Mean (± SD), *n* (%)	Mean (± SD), *n* (%)	Mean (± SD), *n* (%)	
Patient demographics				
Number of complicating comorbidities present^a^				0.301
0	9 (12.9%)	3 (7.5%)	6 (20%)	
1–2	36 (51.4%)	22 (55%)	14 (46.7%)	
≥ 3	25 (35.7%)	15 (37.5%)	10 (33.3%)	
Age (years)	60.4 (± 14.0)	64.4 (± 12.5)	55.1 (± 14.4)	**0.005**
Male sex	35 (50%)	20 (50%)	15 (50%)	0.999
BMI (kg/m^2^)	29.1 (± 5.1)	29.0 (± 4.4)	29.0 (± 5.9)	0.889
Smoking status				0.563
Active	13 (18.6%)	8 (20%)	5 (16.7%)	
Stopped	34 (48.6%)	21 (52.5%)	13 (43.4%)	
Non smoker	23 (32.9%)	11 (27.5%)	12 (40.0%)	
Diabetes mellitus	12 (17.1%)	7 (17.5%)	5 (16.7%)	0.927
Cardiac disease	14 (20%)	7 (17.5%)	7 (23.2%)	0.764
COPD	11 (15.7%)	10 (25%)	1 (3.3%)	**0.019**
Anticoagulative medication	19 (27.1%)	12 (30%)	7 (23.3%)	0.535
Immunosuppressive medication	4 (5.7%)	2 (5%)	2 (6.7%)	0.999
Previous SSI	47 (67%)	27 (67.5%)	20 (66.7%)	0.941
Hernia and wound characteristics				
Number of complicating hernia characteristics present^b^				**0.048**
0	17 (24.3%)	10 (25%)	7 (23.3%)	
1–2	34 (48.6%)	15 (37.5%)	19 (63.3%)	
≥ 3	19 (27.1%)	15 (37.5%)	4 (13.4%)	
Hernia severity class				0.733
Minor complex	10 (14.3%)	5 (12.5%)	5 (16.7%)	
Moderate complex	26 (37.1%)	14 (35%)	12 (40%)	
Major complex	34 (48.6%)	21 (52.5%)	13 (43.3%)	
Modified VHWG classification, grade				0.190
1	3 (4.3%)	2 (5%)	1 (3.3%)	
2	41 (58.6%)	21 (52.5%)	20 (66.7%)	
3	26 (37.1%)	17 (42.5%)	9 (30.0%)	
HPW classification, stage				0.442
1	8 (11.4%)	3 (7.5%)	5 (16.7%)	
2	35 (50%)	20 (50%)	15 (50%)	
3	27 (38.6)	17 (42.5%)	10 (33.3%)	
Stoma present	11 (15.7%)	9 (22.5%)	2 (6.7%)	0.072
Intestinal fistula(s) present	12 (17.1%)	10 (25%)	2 (6.7%)	**0.044**
Infected mesh present	6 (8.6%)	3 (7.5%)	3 (10.0%)	0.999
Previous abdominal surgeries				0.573
0–2	21 (30%)	14 (35%)	7 (23.3%)	
3–4	19 (27.2%)	10 (25%)	9 (30.0%)	
≥ 5	30 (42.8%)	16 (40%)	14 (46.7%)	
Previous hernia repairs				0.427
0	33 (47.1%)	21 (52%)	12 (40%)	
1	14 (20%)	6 (15%)	8 (26.7%)	
≥ 2	23 (32.9%)	13 (32.5%)	10 (33.3%)	
CDC classification				0.134
1	44 (62.9%)	23 (57.5%)	21 (70.0%)	
2	9 (12.9%)	4 (10%)	5 (16.7%)	
3	10 (14.3%)	9 (22.5%)	1 (3.3%)	
4	7 (10%)	4 (10%)	3 (10%)	
Preoperative botox injections	18 (25.7%)	–	18 (60.0%)	**0.001**
Hernia width (cm), median (range)	10.0 (0–30)	10.0 (0–30)	10.0 (0–21)	0.728
Hernia width ≥ 10 cm	30 (42.9%)	18 (45%)	12 (40%)	0.676
Loss of domain (%), median (range)	10.0 (0–52)	11.0 (0–40)	10.0 (0–52)	0.651
Loss of domain ≥ 20%	9 (12.9%)	7 (17.5%)	2 (6.7%)	0.180

^a^Including: active smoking, BMI > 30, *COPD* cardiac disease, *DM* anticoagulative medication, immunosuppressive medication, previous wound infection

^b^Including: presence of a stoma, intestinal fistula, infected mesh, transverse defect width ≥ 10 cm, loss of domain > 20%, previous hernia repair, concomitant bowel surgery

*p*-value in bold reflects significant level *p*<0.05

### Surgical data

Surgical data are presented in Table 2. Concomitant bowel surgery was performed in 24.3% (17 patients). A CST was performed in 61.4% of patients (43 of 70 patients) with open ACS in 35.7% and TAR in 20% of patients. Biosynthetic mesh only was used 43 (61.4%) times, whereas in 23 (32.7%) an additional biologic mesh, and in four (5.8%) an additional synthetic or hybrid biologic mesh was used.

**Table 2:** Surgical data

	Total group (*n* = 70)	LTD (*n* = 40)	MTD (*n* = 30)	*p *value
	*n* (%)	*n* (%)	*n* (%)	
Concomitant bowel surgery	17 (24.3%)	13 (32.5%)	4 (13.3%)	0.064
Component separation (CST)				0.058
No CST	27 (38.6%)	15 (37.5%)	12 (40.0%)	
Open ACS	25 (35.7%)	18 (45%)	7 (23.3%)	
Endo ACS	4 (5.7%)	3 (7.5%)	1 (3.3%)	
Open TAR	14 (20%)	4 (10%)	10 (33.3%)	
Mesh type				0.076
Biosynthetic only	43 (61.4%)	21 (52.5%)	22 (73.3%)	
Additional strattice	18 (25.7%)	14 (35%)	4 (13.3%)	
Additional XenMatrix	5 (7.0%)	5 (12.5%)	–	
Additional OviTex	2 (2.9%)	–	2 (6.7%)	
Additional Vypro	2 (2.9%)	–	2 (6.7%)	
Biosynthetic mesh location				**0.003**
Retro-rectus (sublay)	52 (74.3%)	24 (60%)	28 (93.3%)	
Intra-abdominal (underlay)	3 (4.3%)	2 (5%)	1 (3.3%)	
Onlay reinforcement of biologic	9 (12.9%)	9 (22.5%)	–	
Inlay reinforcement of biologic	6 (8.6%)	5 (12.5%)	1 (3.3%)	
Major full-thickness skin reconstruction	15 (21.4%)	10 (25%)	5 (16.7%)	0.400
Postoperative incisional NPWT	62 (88.6%)	36 (90%)	26 (86.7%)	0.717
Fascial closure				**0.035**
Anterior + posterior fascia closed	53 (75.7%)	28 (70%)	25 (83.3%)	
Posterior fascia closed only	2 (2.9%)	–	2 (6.7%)	
Bridged repair (anterior nor posterior fascia closed)	15 (21.4%)	12 (30%)	3 (10%)	

*CST* component separation technique, *open* *ACS* anterior component separation (Ramirez), *endo* *ACS* endoscopic anterior component separation technique, *open* *TAR* transversus abdominis release

*p*-value in bold reflects significant level *p*<0.05

### Differences in preoperative and operative data between LTD and MTD biosynthetic mesh

Presence of COPD and intestinal fistula(s) was much higher in the LTD group (25.0% vs 3.3% *p* < 0.019 and 25.0% vs 6.7% *p* < 0.044, respectively), associated with a change in mesh indications over time in our practice. Injection of BTA was performed in 60.0% of patients in the MTD group, and in only 2.4% of the LTD group, associated with the change in practice over time (*p* < 0.001). Biosynthetic mesh positioning differed significantly between groups (*p* < 0.003), relating to differences in hernia complexity among the two groups. Biosynthetic mesh was mostly placed in retromuscular (sublay) position in 93.3% of the MTD group vs. 60% of LTD group. Biosynthetic mesh was used as onlay reinforcement after primary fascial closure in 9 patients (22.5%) of the LTD group versus none in the MTD group.

### Clinical outcomes

Clinical outcomes are presented in Table 3. Overall SSI rate in these CAWR patients was 45.7%. Seventeen of 70 patients (24.3%) had a SSI in direct contact with mesh on CT, suspicious of mesh infection; of which 10 of 40 patients (25.0%) in the LTD group and 7 of 30 patients (23.3%) in the MTD group. Ten of those 17 patients with SSI in direct contact with mesh had the biosynthetic mesh in the retro-rectus position (4 of 10 in the LTD group and 6 of 7 in the MTD group). In 7 of 70 patients (10%) and 7 of 17 meshes (41.2%) with persistent local infection, the mesh had to be removed after a median of 229 days since surgery. All 7 mesh removals occurred in the LTD group and none in the MTD group. The mesh salvage rate after in direct contact with SSI was 58.8% (30% in the LTD group and 100% in the MTD group).

**Table 3:** Clinical outcomes

	Total group (*n* = 70)	LTD (*n* = 40)	MTD (*n* = 30)
	*n* (%)	*n* (%)	*n* (%)
Suspected mesh infection because of direct contact with SSI on CT^a^	17 (24.3%)	10 (25%)	7 (23.3%)
After CDC 1 or 2 repair	12 (17.1%)	5 (12.5%)	7 (23.3%)
After CDC 3 or 4 repair	5 (7.1%)	5 (12.5%)	–
Suspected mesh infection, mesh position	17 (24.3%)	10 (25%)	7 (23.3%)
Retro-rectus (sublay)	10 (14.2%)	4 (10%)	6 (20%)
Intra-abdominal (underlay)	2 (2.9%)	2 (5%)	–
Onlay reinforcement of biologic	2 (2.9%)	2 (5%)	–
Inlay reinforcement of biologic	3 (4.3%)	2 (5%)	1 (3.3%)
Removal of mesh	7 (10%)	7 (17.5%)	–
After CDC 1 or 2 repair	4 (5.7%)	4 (10%)	
After CDC 3 or 4 repair	3 (4.3%)	3 (7.5%)	
Removal of mesh according to position	7 (10%)	7 (17.5%)	–
Retro-rectus (sublay)	3 (4.3%)	3 (7.5%)	
Intra-abdominal (underlay)	2 (2.9%)	2 (5%)	
Onlay reinforcement of biologic	1 (1.4%)	1 (2.3%)	
Inlay reinforcement of biologic	1 (1.4%)	1 (2.3%)	
Days between surgery and mesh removal (median, range)	229 (77–895)	229 (77–895)	–
Salvage rate of suspected infected meh	10/17 (58.8%)	3/10 (30%)	7/7 (100%)
SSO			
No SSO	31 (44.3%)	14 (35%)	17 (56.7%)
SSI only	25 (35.7%)	14 (35%)	11 (36.7%)
Wound dehiscence only	3 (4.3%)	3 (7.5%)	–
Seroma/hematoma only	4 (5.7%)	2 (5%)	2 (6.7%)
Combination of different SSOs	7 (10%)	7 (17%)	–
SSI (according to CDC)			
No SSI	38 (54.3%)	19 (47.5%)	19 (63.3%)
Superficial SSI	16 (22.9%)	11 (27.5%)	5 (16.7%)
Deep SSI	2 (2.9%)	1 (2.3%)	1 (3.3%)
Organ space SSI	9 (12.8%)	4 (10%)	5 (16.7%)
Combination of SSIs	5 (7.1%)	5 (12.5%)	–
SSI according to mesh use			
Biosynthetic use only	19/43 (44.2%)	11/21 (52.4%)	8/22 (36.4%)
Additional other mesh	13/27 (48.1%)	10/19 (52.6%)	3/8 (37.5%)
Non-mesh-related reoperations			
Surgical treatment of SSO^b^	9 (12.9%)	6 (15%)	3 (10.0%)
Anastomotic leakage	1 (1.4%)	–	1 (3.3%)
Bowel perforation	1 (1.4%)	1 (2.5%)	–
Preexisting diaphragm paralysis	1 (1.4%)	1 (2.5%)	–
Recurrent hernia repair	2 (2.9%)	2 (5%)	–
Hospital stay (days)			
Median, range	7 (4–72)	9 (4–72)	6 (5–40)
Follow-up (months)			
Median, range	20 (3–46)	35 (13–46)	11 (3–17)
Hernia recurrence	7 (10%)	4 (10%)	3 (10%)
Suspected mesh infection^a^ prior to recurrence	3	2	1
Mesh removal prior to recurrence	2	2	–

^a^Suspected mesh infection defined as biosynthetic mesh in direct contact with SSI on CT

^b^Including surgical treatment of deep SSI (*n* = 4), V.A.C. VeraFlo™ treatment of deep SSI (*n* = 2), evacuation of hematoma (*n* = 1), excision of infected mitek anchors/osteitis (*n* = 1), recurrent enterocutaneous fistula (*n* = 1)

At a median follow-up of 20 months (range 3–46), 7 of 70 patients (10%) had a CT-confirmed recurrent hernia. Median follow-up was 35 months for the LTD group and 11 months for the MTD group. Four of 40 patients (10%) had a recurrence in the LTD group and three of 30 patients (10%) in the MTD group. Two additional patients in the MTD reported symptoms that indicated a possible recurrence. Three of the seven recurrences occurred in patients with SSI in persistent and direct contact with mesh (two patients in the LTD group and one in the MTD group; in the two LTD patients, the mesh needed to be removed).

The cumulative recurrence rate at 13 months postoperative (time at which 1/3 of MTD cohort was still at risk for recurrence) was not statistically different between both groups (*p* = 0.792; see Online supplementary material).

## Discussion

The importance of mesh reinforcement to decrease recurrence rates has been widely acknowledged. However, which mesh to use in which type of patients remains an ongoing subject of discussion [25]. Because patient and hernia characteristics greatly affect clinical outcome and the risk of postoperative wound complications, just as meshes can do, it is difficult to assess the influence of a specific mesh.

Mesh infection with the subsequent need of mesh removal is a feared complication of CAWR. It is, therefore, important to know how the mesh behaves in postoperative local infections; can it be salvaged or does it need to be removed?

Present study evaluates clinical outcomes of two independent series of 70 consecutive patients in total, with moderate to highly complex incisional hernias who underwent single-stage open CAWR using LTD (*n* = 40) or MTD (*n* = 30) biosynthetic mesh. With a postoperative SSI rate of 45.7% and mesh in direct contact with SSI, suspicious of mesh infection, occurring in 24.3% of patients, present study shows that when using biosynthetic meshes in complex patients, short-term outcomes remain challenging. Mesh was removed in 10% of patients because of persistent local infection, giving a salvage rate of nearly 60%. The hernia recurrence rate was 10% at a median follow-up of 20 months.

Comparison with other studies with respect to mesh infection is difficult as studies use various definitions for mesh infections. We were very strict in our evaluation of mesh behavior in infected environment and defined mesh infection as CT- and culture-confirmed direct contact of mesh with SSI. With comparable incidence in postoperative mesh infection in both biosynthetic mesh groups, it is interesting to see that the need for mesh removal for persistent infection occurred in 7 of 70 patients (10%), all in the LTD group (seven out of ten infected meshes). It seems that when LTD mesh comes into contact with an infection, few meshes can withstand this and can be salvaged.

The higher rate of SSI could be explained by the percentage of patients (67%) with previously experienced SSI, which compared to most studies was at least twice as high (if reported at all) [9, 10, 12, 14]. This line of reasoning was also confirmed by the fact that we found that suspected mesh infection also occurred in initially non-contaminated setting. The rate of SSIs in the present study is higher in comparison with other studies using just Phasix™ [9–11, 14, 26–29] or just BIO-A^®^ [12, 30–32], in which the SSI rates ranged 0–17% and 6.2–18.3%, respectively. Sahoo et al. compare different biosynthetic meshes (i.e., Phasix^™^, BIO-A^®^, and Tiger^™^) with synthetic mesh, in clean-contaminated and contaminated wounds and report a SSI rate of 22.4%. [33] In the biosynthetic group, no mesh infection is reported and only one mesh removal (1.7%). Another retrospective study with multiple biosynthetic meshes reports 32.7% SSIs; mesh removal in 3.6% [34]. Another factor affecting SSI rate is the use of open ACS, which is with 35.7% considerably higher than the above-mentioned studies of biosynthetic mesh use (range 0–20%). Open component separation (ACS) with its larger wound beds is known to be highly associated with wound complications [35]. Figure 1 shows that endo-CST but in particular TAR was more frequently used after 2018. In addition, the introduction of preoperative botulinum toxin A in 2018 and its liberal use in our institute was able to prevent the need of CST to reach medial fascial closure. Second, for large defects, the simultaneous introduction of a hybrid biological mesh (Ovitex^®^ reinforced tissue matrix) in our practice caused a shift from extended open anterior CST to prevent bridging repair to use of intra-abdominal underlay hybrid reinforced mesh. These two more recent advances in our practice of CAWR simplified the procedure and reduced the surgical wound surface needed for the repair. Furthermore, our vigorously complication registration and culture sampling could account for our high rate of SSIs in which we register even the smallest wound complication. We also investigated if the use of an additional biologic mesh could be an explanation since the use of biologic mesh is reported with high rates of SSI [36]. In the present series, however, SSI rate was comparable for the use of a single biosynthetic mesh or a biosynthetic combined with biologic mesh.

The recurrence rate was 10% with a median follow-up of 20 months. In the MTD group, two patients reported symptoms indicating a recurrence. Due to COVID-19, we did not invite these patients to the outpatient department and could not confirm a recurrence. The reported recurrence rate for MTD is, therefore, possibly underestimated, and may be 16.7% (5 of 30) instead of 10% (3 of 30). Recurrence rates were comparable with other studies (ranging from 0 to 17%), although not all previously published studies used CT for assessing recurrence so the true numbers could be higher than currently published [9–13, 28–33]. For the LTD biosynthetic mesh group, the median follow-up of 35 months is one of the longest in the literature. For the MTD biosynthetic mesh group, the median follow-up of 11 months in present series is, in our opinion, too short to draw any long-term conclusions on recurrences. It is of note though that the MTD mesh is completely absorbed within 7 months [12], but recurrence rates may still increase as time passes. Three of the seven recurrences occurred in patients with SSI in persistent and direct contact with mesh as shown by CT (2 patients in the LTD group and 1 in the MTD group; in the 2 LTD patients the mesh needed to be removed). It seems that LTD biosynthetic mesh can provide an adequate durable repair, but when it comes into direct contact with a local postoperative infection, the mesh needs to be removed more often than it can be salvaged. Whether or not MTD biosynthetic mesh provides a durable repair needs to be awaited due to the relatively short follow-up period, but our results show that this mesh can be salvaged in direct presence of an infection.

## Strengths and limitations

Although performing a retrospective analysis, which induces possible selection bias, we included all consecutive and prospectively registered patients treated with biosynthetic mesh without any exclusion criteria. Therefore, the results of our study reflect actual clinical outcomes of the use of these biosynthetic meshes in daily surgical practice of a CAWR tertiary referral center. Where more data are needed assessing long-term outcomes of biosynthetic mesh, the LTD cohort in our study has a median follow-up of 34 months, which is relatively long to earlier studies.

The biggest limitation of the present study is that data from both groups are not suitable for direct statistical comparison and should, therefore, be regarded as two independent series of consecutive patients.

Due to the heterogeneous nature of the referred CAWR population, a combination of meshes and techniques was sometimes needed for the CAWR. Exclusion of these patients would not reflect daily practice, and splitting the cohort would result in small and fragmented subgroups making it hard to draw any conclusions.

## Conclusion

Comorbid patients undergoing open complex abdominal wall repair are at high risk of postoperative wound complications regardless of which type of biosynthetic mesh is used. When in persistent and direct contact with infection, LTD biosynthetic meshes may need to be removed, whereas MTD biosynthetic meshes can be salvaged. Present study was not set-up as comparative in nature or to determine which type of biosynthetic mesh should be used in which type of patient, but merely aims to show clinical outcomes of their use in daily practice. It also shows that patient factors are extremely important and that highly complex patients, and not just level of contamination, can equally affect outcomes.

## Supplementary Information

Below is the link to the electronic supplementary material.

Supplementary file1 (DOCX 126 kb)
