# Supplementary figures and images for: Outcomes of mid-term and long-term degradable biosynthetic meshes in single-stage open complex abdominal wall reconstruction
Source: Hernia. 2021 Jun 7;25(6):1647–57. doi: 10.1007/s10029-021-02415-7 (PMC8182350; doi:10.1007/s10029-021-02415-7)

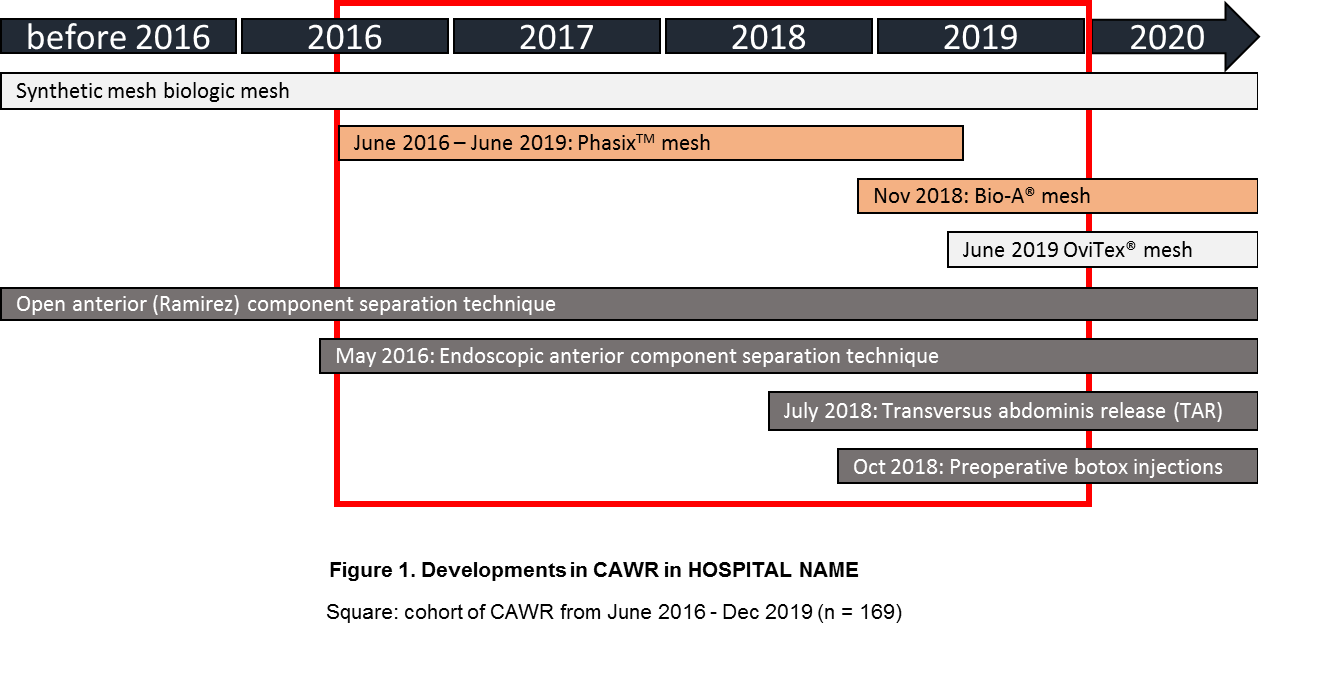


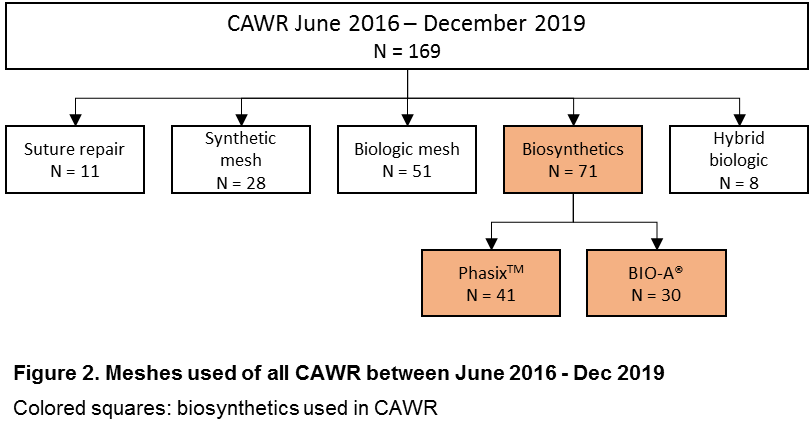


**
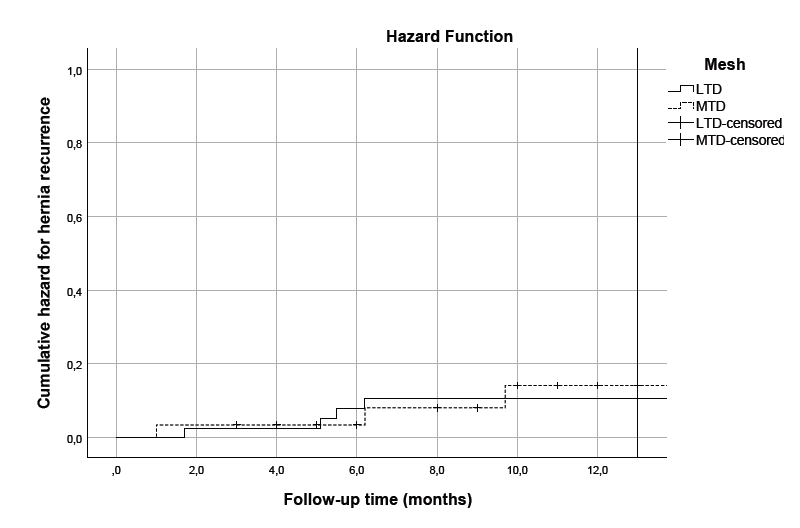
Appendix Figure S1**

**Appendix Table S1**


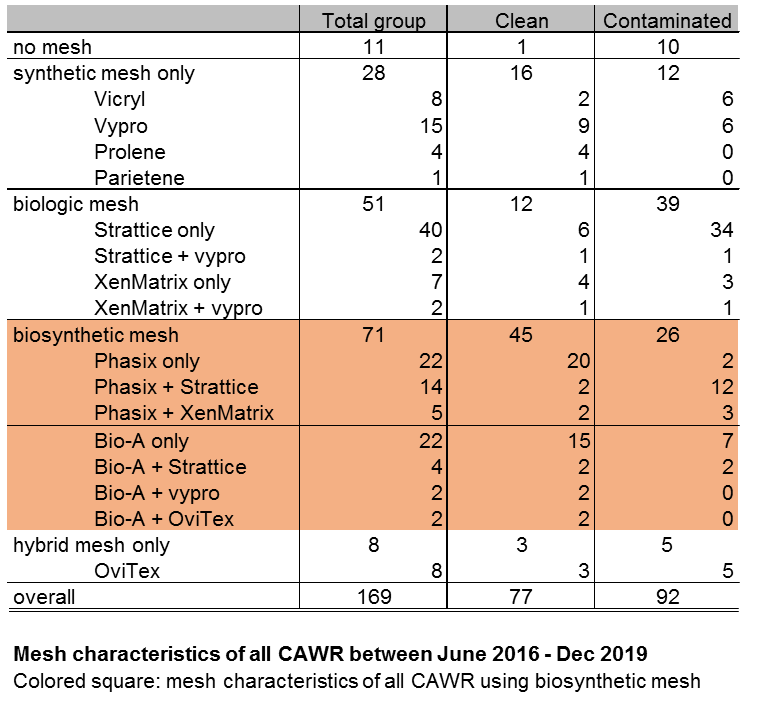

Supplement: Supplementary file 1 — Supplementary file1 (DOCX 126 kb) [file 10029_2021_2415_MOESM1_ESM.docx]
